# Factors associated with intracapsular versus extracapsular hip fracture patterns in elderly patients following low-energy falls: a retrospective analysis from a single tertiary-care center in China

**DOI:** 10.3389/fmed.2026.1753069

**Published:** 2026-09-14

**Authors:** Si-Jia Xia, Zhong-Shuai Sun, Wei-Xin Qiao, Yong Wang, Guo-Chun Zha

**Affiliations:** 1 Department of Orthopaedic Surgery, The Affiliated Hospital of Xuzhou Medical University, Xuzhou, Jiangsu, China; 2 Department of Orthopedics, The First People’s Hospital of Shangqiu, Shangqiu, China; 3 Department of Orthopedic Surgery, The Affiliated Yixing Hospital of Jiangsu University, Wuxi, Jiangsu, China; 4 Regenerative Medicine & Tissue Engineering Research Center, Institute of Medical Innovation and Translation, The Affiliated Yixing Hospital of Jiangsu University, Yixing, China

**Keywords:** elderly, extra-capsular, hip fracture, intra-capsular, low-energy falls, risk factors

## Abstract

**Background:**

Hip fractures represent a significant clinical and public health challenge in the elderly, particularly as populations age globally. While low-energy falls are the primary mechanism of injury, the specific risk factors differentiating intra-capsular and extra-capsular hip fractures remain inadequately characterized. Existing research has largely focused on general fracture risks, leaving a gap in understanding how geometric, nutritional, and metabolic parameters distinctly influence fracture type. Elucidating these differences is essential for advancing personalized prevention in vulnerable aging populations.

**Objectives:**

This study aimed to identify and compare risk factors associated with intra-capsular and extra-capsular hip fractures among elderly patients following low-energy falls.

**Methods:**

A retrospective analysis was conducted involving 636 patients aged 70 years or older with hip fractures. Demographic characteristics, geometric parameters (including neck-shaft angle, cortical index, and Singh index), and biochemical markers (hemoglobin, albumin, total protein, and fasting blood glucose) were evaluated. Multivariable logistic regression was employed to identify predictors of fracture type.

**Results:**

Patients with extra-capsular fractures were significantly older and exhibited lower hemoglobin levels, albumin, total protein, cortical index, and neck-shaft angles, along with higher fasting blood glucose compared to those with intra-capsular fractures (all *p* < 0.05). The logistic regression model identified cortical index, neck-shaft angle, hemoglobin, and fasting blood glucose as significant independent predictors of fracture type.

**Conclusion:**

Extra-capsular hip fractures are associated with poorer nutritional status, reduced bone density, smaller neck-shaft angle, and higher blood glucose levels. These findings suggest that extra-capsular fractures are differentially associated with poorer nutritional status, reduced cortical thickness, smaller neck-shaft angles, and higher glucose levels. Our results provide preliminary evidence that combined assessments may aid in risk stratification; however, these associations warrant confirmation in prospective cohort studies before clinical translation.

## 1 Introduction

The global aging population has led to a rising incidence of hip fractures among the elderly, posing a significant challenge to orthopedic trauma care (1, 2). These fractures, often resulting from low-energy mechanisms such as falls, are frequently associated with osteoporosis (3, 4). Anatomically, hip fractures are classified as intra-capsular (femoral neck) or extra-capsular (intertrochanteric or subtrochanteric), each with distinct treatment pathways and prognoses (5, 6).

Although the injury mechanism is often similar, the factors predisposing an individual to a specific fracture type remain inadequately explored. Previous studies suggest that proximal femoral geometry, such as neck-shaft angle (NSA) and fat distribution, may influence fracture patterns (7, 8). However, the roles of nutritional status (e.g., hemoglobin, albumin, total protein) and metabolic factors (e.g., diabetes mellitus, fasting blood glucose) are less clear.

This study aims to: (1) identify and compare demographic, geometric, and biochemical factors associated with intra-capsular versus extra-capsular hip fractures in elderly patients following low-energy falls; (2) establish a multivariable logistic regression model to discern significant predictors of fracture type; and (3) provide insights into the clinical differentiation and underlying mechanisms of these two fracture patterns based on the identified risk factors.

## 2 Methods

### 2.1 Study population and design

A retrospective analysis was conducted on patients admitted with hip fractures to the Affiliated Hospital of Xuzhou Medical University between January 2010 and December 2020.

This retrospective study included patients aged 70 years or older who presented with a fresh, unilateral hip fracture (either intracapsular or extracapsular) confirmed by anteroposterior and lateral pelvic x-rays or computed tomography (CT) scans. All fractures were caused by low-energy trauma, strictly defined as a fall from standing height or less (e.g., slipping on level ground, falling from a chair/bed), excluding traffic accidents or falls from stairs.

Patients were included if they had complete admission records of demographic data, standard biochemical panels, and pelvic imaging necessary for geometric measurements.

Exclusion criteria were: (1) pathological fractures secondary to primary bone tumors or metastatic lesions (confirmed by MRI or biopsy); (2) pre-existing lower limb deformities (e.g., severe knee/hip osteoarthritis with varus/valgus >15°, or previous malunion) that could alter biomechanical loading; (3) multiple fractures (e.g., concomitant vertebral or pelvic ring fractures) or severe comorbidities (defined as American Society of Anesthesiologists [ASA] grade ≥ IV, or end-stage organ failure affecting independent mobility); (4) cognitive impairment (defined as a documented clinical diagnosis of dementia, or a Mini-Mental State Examination [MMSE] score < 24 on admission, making the fall mechanism unreliable or history taking invalid).

All laboratory samples (hemoglobin, albumin, total protein, and fasting blood glucose) were drawn within 24 hours of admission, prior to any intravenous fluid resuscitation, to avoid hemodilution bias.

### 2.2 Data collection

Data collected included: age, sex, body mass index (BMI), neck-shaft angle (NSA), acetabular abduction and anteversion angles, cortical index Singh Index, hemoglobin (Hb), albumin, total protein, fasting blood glucose (FBG), lymphocyte count (LC) and percentage (LP), history of diabetes mellitus (DM). The general details of the patients are shown in Table 1.

**Table 1:** Comparison between intra-capsular and extra-capsular fracture groups.

Variable	Intra-capsular (*n* = 334)	Extra-capsular (*n* = 302)	*P*-value
Age (years)	79.10 ± 6.10	80.96 ± 5.62	**<0**.**001**
BMI (kg/m^2^)	26.18 ± 2.85	26.25 ± 2.82	0.749
Sex (*n*, %)			0.059
Male	94 (28.14%)	106 (35.1%)	
Female	240 (71.86%)	196 (64.9%)	
Singh index (*n*, %)			0.854
Ⅰ	56 (16.76%)	52 (17.21%)	
Ⅱ	161 (48.20%)	126 (41.72%)	
Ⅲ	87 (26.04%)	90 (29.80%)	
Ⅳ	12 (3.59%)	23 (7.61%)	
Ⅴ	15 (4.49%)	8 (2.64%)	
Ⅵ	3 (0.92%)	3 (1.02%)	
Cortical Index	0.523 ± 0.08	0.493 ± 0.08	**<0**.**001**
Abduction angle (°)	43.08 ± 5.99	43.34 ± 6.04	0.577
Anteversion angle (°)	24.78 ± 9.46	23.78 ± 9.21	0.287
Neck-Shaft Angle (°)	141.10 ± 9.75	138.80 ± 10.08	**0**.**001**
Lymphocyte percentage (%)	17.00 ± 7.55	15.54 ± 5.49	0.125
Lymphocyte count (×10⁹/L)	1.29 ± 0.78	1.28 ± 0.48	0.391
Hemoglobin (g/L)	120.64 ± 18.03	105.82 ± 17.21	**<0**.**001**
Albumin (g/L)	38.42 ± 4.64	37.17 ± 4.02	**<0**.**001**
Total Protein (g/L)	67.04 ± 6.71	64.64 ± 6.40	**<0**.**001**
Fasting Glucose (mmol/L)	6.24 ± 2.12	6.94 ± 2.75	**<0**.**001**
Diabetes Mellitus (*n*, %)	72 (21.6%)	117 (38.7%)	**<0**.**001**

Bold *P*-values indicate statistically significant results.

### 2.3 Statistical analysis

Statistical analyses were performed using IBM SPSS Statistics v22. Continuous variables are presented as mean ± standard deviation and compared using Student's t-test or Mann–Whitney U test. Categorical variables are presented as n (%) and compared using the Chi-square test. Inter-observer reliability was assessed using Intraclass Correlation Coefficients (ICC). Multivariable logistic regression (backward stepwise method) was used to identify independent predictors of fracture type, with results expressed as odds ratios (OR) and 95% confidence intervals (CI). A p-value < 0.05 was considered statistically significant.

## 3 Results

### 3.1 Comparison between fracture types

A comparison of variables between the two fracture groups is shown in Table 1. Patients with extra-capsular fractures were significantly older and had significantly lower hemoglobin, albumin, total protein, cortical index, and neck-shaft angle, but higher fasting blood glucose and a higher prevalence of diabetes (all *P* < 0.001). No significant differences were found in BMI, sex distribution, Singh index, or acetabular angles.

### 3.2 Multivariable logistic regression analysis

Logistic regression analysis (Table 2) identified several independent predictors. A lower cortical index (OR: 0.013, *p* < 0.001), smaller neck-shaft angle (OR: 0.979, *p* = 0.019), lower hemoglobin (OR: 0.955, *p* < 0.001), and higher fasting blood glucose (OR: 1.159, *p* < 0.001) were significantly associated with an increased likelihood of extra-capsular fracture.

**Table 2:** Multivariable logistic regression analysis for predictors of fracture type (extra- vs. intra-capsular).

Variable	Adjusted OR (95% CI)	*P*-value
Cortical Index	0.013 (0.001–0.120)	**<0**.**001**
Neck-Shaft Angle	0.979 (0.961–0.998)	**0**.**019**
Hemoglobin	0.955 (0.943–0.967)	**<0**.**001**
Fasting Blood Glucose	1.159 (1.064–1.253)	**<0**.**001**

Bold *P*-values indicate statistically significant results.

### 3.3 ROC analysis

As shown in the table (Table 3), the AUC values for individual parameters (cortical index: 0.619; neck-shaft angle: 0.576) indicate modest discriminatory ability, while the combined model demonstrated improved performance (AUC: 0.656).

**Table 3:** ROC analysis.

Variable	AUC (95%CI)	Cutoff	95% CI	*P*-value
Neck-Shaft Angle	0.576	134.9500	0.531–0.620	**0**.**001**
Cortical Index	0.619	0.5145	0.575–0.662	**<0**.**001**

Bold *P*-values indicate statistically significant results.

## 4 Discussion

This study provides a comprehensive analysis of factors differentiating intra-capsular from extra-capsular hip fractures in an elderly Chinese population. Our key findings indicate that fracture type is associated with a combination of geometric, nutritional, and metabolic factors, rather than a single parameter. These insights not only enhance our pathophysiological understanding but also carry significant implications for clinical risk stratification and personalized management.

Our study confirmed the role of proximal femoral geometry. A smaller neck-shaft angle was independently associated with extra-capsular fractures. This aligns with the principle of stress shielding and provides a mechanical explanation for the clinical observation (9, 10). Conversely, a smaller NSA may shift the stress distribution towards the intertrochanteric region, predisposing to extra-capsular fractures (11, 12). The cortical index, a measure of cortical bone thickness, was also a strong independent predictor. A lower index, indicating cortical thinning and osteoporosis, was significantly associated with extra-capsular fractures, which is logical given the greater reliance of the intertrochanteric region on cortical bone for structural integrity (13).

Our analysis highlights the significant influence of systemic patient factors. Nutritional markers, including hemoglobin, albumin, and total protein, were all significantly lower in the extra-capsular fracture group. Hypoalbuminemia has been previously linked to osteoporosis and sarcopenia (14, 15), both of which can contribute to frailty and alter fall mechanics, potentially increasing the risk of a more severe fracture pattern. The significantly lower hemoglobin levels in extra-capsular fractures could be a reflection of greater peri-fracture blood loss into the soft tissues, as these fractures are often less contained by the joint capsule compared to intra-capsular fractures (16).

A novel and strong association was found with metabolic health. Higher fasting blood glucose and a greater prevalence of diabetes were strongly linked to extra-capsular fractures. Diabetes can impair bone quality through various mechanisms, including advanced glycation end-products (AGEs) that compromise collagen strength, leading to “brittle bone” (17). This may make the metaphyseal bone of the intertrochanteric region more vulnerable to failure.

Several limitations should be considered in this study. First, the retrospective design is susceptible to selection bias, and the use of single-center data may restrict the generalizability of the findings. Additionally, the adoption of subjective radiographic assessments, such as the Singh index, might compromise diagnostic accuracy. Beyond imaging, key metabolic parameters—including vitamin D and bone turnover markers—were not comprehensively evaluated, and potential unmeasured confounders such as muscle mass and detailed fall history may further influence the results. Moreover, the cross-sectional nature of the analysis impedes causal inference, particularly since nutritional markers could reflect changes following fracture rather than predisposing factors. Finally, as the data originated from a Chinese cohort, the results may not be readily generalizable to other ethnic or geographic populations. Future prospective, multi-center studies incorporating advanced imaging, comprehensive biochemical profiling, and functional assessments are warranted to validate these findings and establish causal relationships.

## 5 Conclusion

In this retrospective cohort of 636 elderly patients with low-energy hip fractures, extra-capsular fractures were independently associated with lower cortical index, narrower neck-shaft angle, reduced hemoglobin levels, and elevated fasting glucose levels. These associations suggest that a combination of geometric bone properties, femoral morphology, nutritional status, and gluco-metabolic regulation may contribute to the differentiation of intracapsular and extracapsular fracture patterns. However, given the retrospective design and the post-fracture timing of laboratory measurements, these findings should be interpreted as hypothesis-generating rather than as clinically validated predictors or causal determinants. The results provide a rationale for future prospective multicenter studies with standardized pre-fracture biomarker and functional assessments, which are needed to confirm these associations and to develop validated risk scores. Until such validation is available, translation of these findings into routine clinical practice is not yet warranted.

## Data Availability

The datasets used or analyzed during the current study are available from the corresponding author on reasonable request.
